# CREB-dependent LPA-induced signaling initiates a pro-fibrotic feedback loop between small airway basal cells and fibroblasts

**DOI:** 10.1186/s12931-021-01677-0

**Published:** 2021-04-01

**Authors:** Shyam Nathan, Haijun Zhang, Mirko Andreoli, Philip L. Leopold, Ronald G. Crystal

**Affiliations:** Department of Genetic Medicine, Weill Cornell Medical College, 1300 York Avenue, Box 164, New York, NY 10065 USA

**Keywords:** Lysophosphatidic acid, Airway basal cell, CREB, Fibroblast, Intracellular signaling, Idiopathic pulmonary fibrosis, Lung, Autotaxin, COL1A1, ACTA2

## Abstract

**Background:**

Lysophosphatidic acid (LPA), generated extracellularly by the action of autotaxin and phospholipase A2, functions through LPA receptors (LPARs) or sphingosine-1-phosphate receptors (S1PRs) to induce pro-fibrotic signaling in the lower respiratory tract of patients with idiopathic pulmonary fibrosis (IPF). We hypothesized that LPA induces changes in small airway epithelial (SAE) basal cells (BC) that create cross-talk between the BC and normal human lung fibroblasts (NHLF), enhancing myofibroblast formation.

**Methods:**

To assess LPA-induced signaling, BC were treated with LPA for 2.5 min and cell lysates were analyzed by phosphokinase array and Western blot. To assess transcriptional changes, BC were treated with LPA for 3 h and harvested for collection and analysis of RNA by quantitative polymerase chain reaction (qPCR). To assess signaling protein production and function, BC were washed thoroughly after LPA treatment and incubated for 24 h before collection for protein analysis by ELISA or functional analysis by transfer of conditioned medium to NHLF cultures. Transcription, protein production, and proliferation of NHLF were assessed.

**Results:**

LPA treatment induced signaling by cAMP response element-binding protein (CREB), extracellular signal-related kinases 1 and 2 (Erk1/2), and epithelial growth factor receptor (EGFR) resulting in elevated expression of connective tissue growth factor (*CTGF*), endothelin-1 (*EDN1*/ET-1 protein), and platelet derived growth factor B (*PDGFB*) at the mRNA and protein levels. The conditioned medium from LPA-treated BC induced NHLF proliferation and increased NHLF expression of collagen I (*COL1A1*), smooth muscle actin (*ACTA2*), and autotaxin (*ENPP2*) at the mRNA and protein levels. Increased autotaxin secretion from NHLF correlated with increased LPA in the NHLF culture medium. Inhibition of CREB signaling blocked LPA-induced changes in BC transcription and translation as well as the pro-fibrotic effects of the conditioned medium on NHLF.

**Conclusion:**

Inhibition of CREB signaling may represent a novel target for alleviating the LPA-induced pro-fibrotic feedback loop between SAE BC and NHLF.

**Supplementary Information:**

The online version contains supplementary material available at 10.1186/s12931-021-01677-0.

## Introduction

Idiopathic pulmonary fibrosis (IPF) is a chronic, lung disorder of unknown etiology characterized by the development of fibrosis of the alveoli and small airways, leading to progressive decline of respiratory function with a high morbidity and mortality [1–6]. A key feature of IPF is the increase number of lung interstitial fibroblasts, with an increase in production of collagen, and fibroblast differentiation into myofibroblasts [2, 7–9]. The significant increase in extracellular matrix deposition and myo-fibroblast differentiation results in “fibrotic foci,” histologically observed as an abnormal collections of fibroblasts and epithelial cells in the alveolar interstitium and airspaces [2, 8] and the walls of the small airways [1, 10]. Expression levels of pro-fibrotic growth factors such as transforming growth factor beta 1 (TGFB1), connective tissue growth factor (CTGF), endothelin 1 (ET-1), and platelet derived growth factor (PDGF) among other mediators have all been implicated in the pathogenesis of IPF [2, 5, 11–15].

Small airway basal cells (BC), the stem/progenitor cells of the airway epithelium, play an important role in the pathogenesis of IPF. BC undergo a form of epithelial to mesenchymal transition (EMT), migrate distally toward alveoli, proliferate, and become enveloped in fibrotic foci [16–18]. RNA seq and single cell gene expression data have identified markers of BC that are co-expressed with alveolar type 2 epithelial cells in IPF including pathways regulating migration, EMT and proliferation [19–21]. Importantly, the aberrant activation of BC in the IPF fibrotic foci produce mediators that contribute to the proliferation and differentiation of the fibroblast/myofibroblast population. These factors include CTGF, ET-1, TGFβ, PDGF, matrix metalloproteinases, tumor necrosis factor and a number of chemokines [6, 22, 23].

The lysophosphatidic acid (LPA) signaling pathway was originally implicated in the pathogenesis of IPF by Tager et al. [24]. LPA is a phosphorylated mono-acyl glycerol derived from enzymatic decomposition of phosphatidylcholine through the actions of phospholipase A2 (PLA2) to remove an acyl chain and autotaxin (ATX) to remove choline from the head group [25]. LPA signals through G protein coupled receptors including LPA receptors 1 through 6 (LPAR1-6) and sphingosine-1-phosphate receptors 1 through 5 (S1PR1-5) [25–27]. LPA and ATX levels are increased in IPF epithelial lining fluid [26], and manipulations of the LPA signaling pathway can modulate lung fibrosis in mouse models [24, 28–31].

To help understand the mechanism by which LPA contributes to the pathogenesis of the fibrosis associated with IPF, we developed an in vitro model focused on BC-fibroblast interactions relevant to the formation and maintenance of fibrotic foci. Primary BC were obtained from healthy nonsmokers and were treated with LPA. Following the treatment, BC increased expression of fibroblast-related growth factors implicated in fibroblast proliferation and elevated collagen expression and myofibroblast transformation. While, LPA-activated CREB, ERK1/2, and EGFR signaling pathways, blockage of only the CREB pathway suppressed the fibroblast response to LPA-induced BC gene expression. Finally, BC-induced changes in fibroblasts included elevated levels of fibroblast expression of ATX leading to elevated levels of LPA in the cell culture medium. Collectively, these observations suggest that BC and fibroblasts participate in a positive feedback loop to maintain elevated levels of LPA that support lung fibrosis.

## Methods

### Small airway epithelial sampling and basal cell isolation

Small airway epithelial cells were collected by fiberoptic bronchoscopy with brushing as previously described [32]. All donors were healthy nonsmokers (see Additional file 1: Table SI). The cells were dislodged from the brushes by flicking the brush tip in 5 ml of ice-cold PneumaCult ExPlus complete medium (StemCell Technologies, Cambridge, MA). The collected airway epithelial cells were pelleted by centrifugation (250 × g, 5 min) and disaggregated by resuspension in 0.05% trypsin-ethylenediaminetetraacetic acid (EDTA; Invitrogen, Carlsbad CA) for 5 min, at 37 °C. Trypsinization was stopped by addition of HEPES buffered saline, (Lonza, Basel, Switzerland) supplemented with 15% fetal bovine serum (FBS; Invitrogen), and the cells were washed once with 5 ml of phosphate buffered saline, pH 7.4 (PBS), at 23 °C and resuspended in 5 ml of in PneumaCult ExPlus complete medium.

To isolate the BC, the epithelial cells (2.5 × 10^5)^ were plated in T25 flasks in 5 ml of ExPlus complete medium and maintained in a humidified atmosphere of 5% CO_2_ at 37 °C. The next day, unattached cells were removed and medium was then changed every 2 days. To passage the cells, the primary BC were treated with trypsin and seeded at 3000 cells/cm^2^ in PneumaCult ExPlus complete medium. The following day, the media was replaced with fresh ExPlus complete medium, and changed every 2 days. Frozen BC cells were kept in stock from passages 1–3. Before plating BC cells, 2 mM of collagen IV (Sigma, St.Louis, MO) solution in molecular grade water (Hyclone, Logan, Utah) was sterile filtered using a Millipore 0.2 µm filter (Sigma) and then added to a cell culture flask and incubated at 37 °C for 1 h. The collagen IV solution was aspirated and the flask washed three times in PBS and sterilized with UV light. After thawing, the BC were centrifuged for 5 min until pelleted. The cells were resuspended in PneumaCult ExPlus complete medium and plated on collagen coated cell culture flasks and grown till 70–80% confluence for experiment use.

### LPA stimulation of small airway basal cells

To study the effect of LPA on BC, the BC were plated in PneumaCult ExPlus complete medium. After 24 h, BC were washed twice with PBS, once with unsupplemented ExPlus medium, and incubated with unsupplemented ExPlus medium in the absence (control) or in the presence of 1.0 µg/ml LPA (Echelon Bioscience, Salt Lake City, UT) for 3 h. After a 3 h incubation, the BC were harvested with Trizol (Invitrogen) for RNA isolation.

### Fibrotic basal cell gene expression

To quantify CTGF, EDN1, TGFB1, and PDGFB gene expression, total RNA in the aqueous phase after Trizol extraction was purified using an RNAEasy MinElute RNA purification kit (Qiagen, Germantown MD). RNA concentration was determined using a NanoDrop ND-100 spectrophotometer (NanoDrop Technologies, Wilmington, DE). First-strand cDNA was synthesized from 0.5 μg of total RNA using TaqMan Reverse Transcription Reagents with random hexamer priming (Applied Biosystems, Foster City, CA). All samples were analyzed in triplicate at a cDNA dilution of 1:10. All reactions were run on an Sequence Detection System 7500 (Applied Biosystems) and relative expression levels determined using the ΔCt method with 18S ribosomal RNA as an endogenous control [33]. Primers included CTGF (Hs00170014_m1), EDN1 (Hs00174961_m1), TGFB1 (Hs00998133_ m1), PDGFB (Hs00966522_m1), and 18 s RNA (Hs99999901_s1) (Applied Biosystems).

Cell culture supernatants/conditioned media harvested from BC were analyzed for protein levels of CTGF, ET-1, TGFB1, and PDGFB. Levels of secreted CTGF, ET-1, TGFB1, and PDGFB were assessed in BC-conditioned medium using human CTGF ABTS ELISA (Peprotech, Rocky Hill, NJ), endothelin-1 human ELISA kit (ThermoFisher Scientific, Waltham, MA), human TGF beta 1 ELISA Kit (Abcam, Cambridge, MA), and PDGF-BB Quantikine ELISA kit (R&D Systems, Minneapolis, MN) according to the manufacturer’s instructions.

### LPA effect on basal cell intracellular signaling

To study the effect of BC stimulated with LPA on phosphorylation of kinases and their substrates, passage 3 basal cells were first plated in PneumaCult ExPlus complete medium in 6 well plates at 2.5 × 10^5^ cells per well. After 24 h, basal cells were washed twice with PBS, once with unsupplemented ExPlus medium, and incubated with unsupplemented ExPlus medium in the absence (control) or presence of LPA (1.0 g$\mu$/ml). After a 2.5 min incubation, the BC were harvested using RIPA Buffer (ThermoFisher) with protease inhibitors (ThermoFisher) and analyzed for total protein concentration using a BCA protein assay (ThermoFisher). Total protein (200 µg) for each nonsmoker BC was used to analyze the protein expression of a variety of phosphorylated kinases and it substrates using Human Phospho-Kinase Array Proteome Profiler (R&D Systems) according to the manufacturer’s instructions. Results were collected by chemiluminscence detection and exposure of photographic film (Denville Scientific, Metuchen, NJ). Quantification of the phosphokinase array was done using ImageJ (NIH; [https://imagej.nih.gov/ij/]) applied to a series of exposures of different times to ensure that the film exposure was in the linear range of detection.

### Western analysis

LPA-induced signaling was further examined by determining the ratio of the phosphorylated form of each protein to the total amount of the protein using Western analysis. Whole cell lysates were separated by 4–12% sodium dodecyl sulfate–polyacrylamide gel electrophoresis (SDS-PAGE) gel and transferred to a PVDF membrane using a transfer apparatus according to manufacturer’s protocol (Bio-Rad, Hercules CA). After incubation with 5% bovine serum albumin (BSA, fraction V; Sigma) in PBST (10 mM sodium phosphate, pH 8, 150 mM NaCl, 0.5% Tween 20) for 60 min, the membrane was rinsed once with PBST and incubated with antibodies against phosphorylated CREB (S133) (1:1000, #9191), phospho-Erk1/2 (Erk1/2; Thr202/Tyr204; 1:1000, #9101) and Erk1/2 (1:1,000, #9102), phospho-p70S6kinase (T389) (1:1000, #9205 and #9234), p70 S6 kinase (T389) (1:1000, #9205 and #9234) (all from Cell Signaling Technology, Danvers MA), phospho-p70 S6 kinase (1:1000, MAB8963, R&D Systems), GAPDH (1:20000, sc47724, Santa Cruz Biotechnology, Dallas, TX) at 4 °C overnight. Membranes were washed three times for 10 min and incubated with 1:5000 dilution of anti-rabbit or anti-mouse secondary antibodies for 1 h. The membrane was washed three times for 15 min with PBS and detected using a chemiluminscent detection system (ECL; Thermo Fisher) using manufacturer’s protocol.

### Effect of signaling pathway inhibitors

To study the effect of signaling inhibitors on LPA-stimulated BC expression of pro-fibrotic growth factors, BC were first plated in PneumaCult ExPlus complete medium. After 24 h, BC were washed twice with PBS, once with unsupplemented ExPlus medium, and incubated for 3 h with unsupplemented ExPlus medium in the absence (control) or presence of LPA (1.0 g$\mu$/ml) $\pm$ signaling inhibitors: CREB inhibitor 666-15 (200 nM; Echelon Biosciences, Salt Lake City, UT), ERK1/2 inhibitor LY3214996 (5.0 M$\mu$; Med Chem Express, Monmouth Junction, NJ), EGFR inhibitor AG1478 (10.0 M$\mu$; CalBiochem, San Diego, CA) For CREB and EGFR inhibitor, 0.005%, or 0.01% DMSO, were added, respectively, as vehicle controls. After incubation, BC cultures were harvested with Trizol for RNA isolation. Quantitative PCR was used to evaluate growth factor expression as described above.

To assess the effect of signaling inhibitors on LPA-stimulated pro-fibrotic protein secretion from BC, cells were first plated in PneumaCult ExPlus complete medium in 6-well plates as described above. After 24 h, BC were incubated with PneumaCult ExPlus complete medium in the absence (control) or presence of LPA (1 g$\mu$/ml) $\pm$ signaling inhibitors as described above. After a 3 h incubation, BC were washed twice with PBS, once with unsupplemented ExPlus medium, and incubated with unsupplemented ExPlus medium. BC were either harvested with Trizol immediately for mRNA analysis as described above, or, after a 24 h incubation, the BC conditioned media/culture supernatants were harvested and evaluated for fibrotic factors via ELISA assay as described above.

### Effect of LPA-stimulated BC conditioned media on fibroblasts

To study the effect of LPA-stimulated BC-conditioned media on normal human lung fibroblast (NHLF) proliferation in the absence (control) or presence of signaling inhibitors for CREB, Erk1/2, or EGFR, NHLF were plated in fibroblast growth media (FGM-2 supplemented with 2% FBS, 0.1% bFGF, 0.1% insulin, 0.1% GA-1000; Lonza). After 24 h, NHLF were washed twice with PBS, once with unsupplemented ExPlus medium, and incubated with unsupplemented PneumaCult ExPlus medium. After another 24 h, NHLF were incubated with 500 µl unsupplemented PneumaCult ExPlus medium (control) or 500 µl of the undiluted LPA-stimulated BC-conditioned media. After 48 h incubation in conditioned medium, the NHLF were trypsinized and counted using trypan blue exclusion.

To study the effect of LPA-stimulated BC-conditioned media on normal human lung fibroblast (NHLF) myofibroblast (ACTA2), collagen I (COL1A1) or autotaxin (ENPP2) gene expression in the absence (control) or presence of signaling inhibitors for CREB, Erk1/2, or EGFR, NHLF were first plated and treated as described above. After 24 h incubation in conditioned medium, NHLF were harvested with Trizol for RNA isolation as described above.

To evaluate the effect of LPA-stimulated BC-conditioned media on the expression and secretion of proteins in NHLF in the absence (control) or presence of signaling inhibitors (described above), NHLF were plated and treated with media as described in the previous paragraph. After a 24 h incubation in conditioned media, the NHLF culture supernatants were harvested and evaluated for collagen I or autotaxin. In order to further characterize autotaxin secretion, levels of LPA, an autotaxin enzymatic product, was measured in the cell culture medium. Collagen I, autotaxin, and LPA levels were determined in NHLF-conditioned medium using ELISA assays. The human COL1A1 ELISA kit (MyBioSource, La Jolla, CA), ENPP2 human ELISA kit (R&D Systems), human LPA ELISA Kit (Echelon Biosciences) were performed according to the manufacturer’s instructions.

To study the effect of LPA-stimulated BC-conditioned media on NHLF expression of smooth muscle actin (ACTA2), after plating and treatment of NHLF with LPA-stimulated BC-conditioned media for 24 h as described above, NHLF were harvested by lysis as described above. ACTA2 protein levels were determined using an ELISA assay for human ACTA2 (Abcam) according to the manufacturer’s instructions.

### Statistical analysis

Statistical comparisons were calculated using an unpaired two-tailed Student’s t-test with equal variance and nested ANOVA using GraphPad Prism software (GraphPad Software, San Diego, CA) where p < 0.05 was considered significant.

### Study approval

Airway epithelial cell samples were collected from normal, healthy non-smoker volunteers following written informed consent with approval from the Weill Cornell Medical College Institutional Review Board (IRB) pursuant to Protocol #0905010391 entitled, “The Natural History of Gene Expression in Lung Cells of Non-Smokers, Smokers, and Ex-Smokers in Health and Disease.”

## Results

### LPA induces expression of pro-fibrotic growth factors in basal cells

**Fig. 1:**
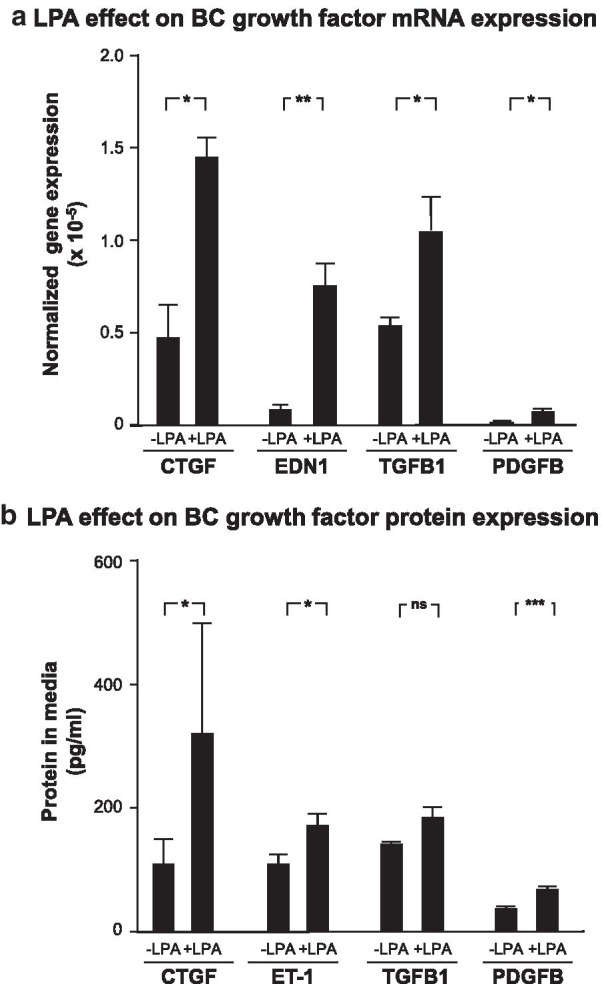
Effect of lysophosphatidic acid (LPA) on fibrotic growth factor expression in the small airway epithelial basal cells (SAE BC). Primary SAE BC from each of 3 non-smoking individuals were plated in triplicate in the presence or absence of 1.0 μg/ml LPA and evaluated for expression of connective tissue growth factor (*CTGF*), endothelin-1 (*EDN1*/ET-1), transforming growth factor beta (*TGFB1*), and platelet derived growth factor family member B (*PDGFB*). **a** mRNA. After a 3 h LPA exposure, RNA was harvested and evaluated by qRT-PCR normalized to 18S RNA. **b** Protein. After a 3 h LPA exposure, LPA was removed and cells were incubated with basal media without growth factor supplements for 24 h. Conditioned media was harvested and evaluated for growth factor content by ELISA. Each sample was assessed in triplicate; data are expressed as the mean value of the 3 donors ± SE. *p < 0.05, **p < 0.01, ***p < 0.001

In order to determine the response of BC to LPA exposure, BC were treated with 1 µg/ml LPA for 3 h. After the treatment, total RNA was collected and analyzed by quantitative PCR for expression of fibrosis-related genes. Exposure to 1 μg/ml LPA led to an increase in *CTGF* mRNA levels of approximately threefold, an increase of greater than a fivefold in *EDN1* expression, a 1.2-fold increase in *TGFB1* expression, and a tenfold increase in *PDGFB* expression (Fig. 1a). To determine whether increases in mRNA levels translated into higher levels of expressed pro-fibrotic growth factor proteins, SAE BC cell culture supernatant was collected 24 h after completion of the 3 h LPA treatment and assessed for levels of the specific proteins by ELISA. Consistent with the findings in the mRNA expression data, CTGF, ET-1, and PDGFB levels were significantly increased in the cell culture medium following LPA treatment (Fig. 1b). TGFB1 levels, while showing an upward trend, were not significantly increased.

Prior to examining the effect of LPA on BC, a dose–response experiment was performed in order to determine the concentration of LPA to be used for treatment of small airway epithelial BC. A range from 0.1 µg/ml to 10 µg/ml was tested for the ability to change expression levels of CTGF, EDN1, TGFB1, PDGFA, PDGFB, and PDGFC (Additional file 1: Figure S1). A concentration of 1 µg/ml LPA was chosen as the concentration for treatment based on dose–response results showing that 1 µg/ml LPA led to significant increases mRNA levels of all six target mRNAs while only three out of six were elevated by 0.1 µg/ml LPA. Raising the concentration of LPA to 10 µg/ml did not significantly change the levels of target mRNAs compared to 1 µg/ml LPA. The concentration of LPA previously reported in bronchoalveolar lavage fluid from IPF patients ranged from 6 to 22 nM [24]. Due to the fact that bronchoalveolar lavage fluid represents a dilution of epithelial lining fluid by a factor of approximately 100 [34], the actual concentration of LPA in epithelial lining fluid of IPF patients is estimated to range from 0.6 to 2.2 µM. In comparison, the LPA concentration selected in the dose response (1 µg/ml) corresponds to a concentration of 2.4 µM. *PDGFA, PDGFB, and PDGFC* transcript levels were all increased significantly by 1 µg/ml LPA (Additional file 1: Figure S1). In contrast, the *PDGFD* transcript was not detectable by quantitative PCR. *PDGFB* was chosen as representative of this group of genes to be included for further analysis since *PDGFB* transcripts exhibited the greatest response (fold-change) in response to LPA treatment.

### LPA activation of BCs induces CREB, ERK1/2, and EGFR phosphorylation

**Fig. 2:**
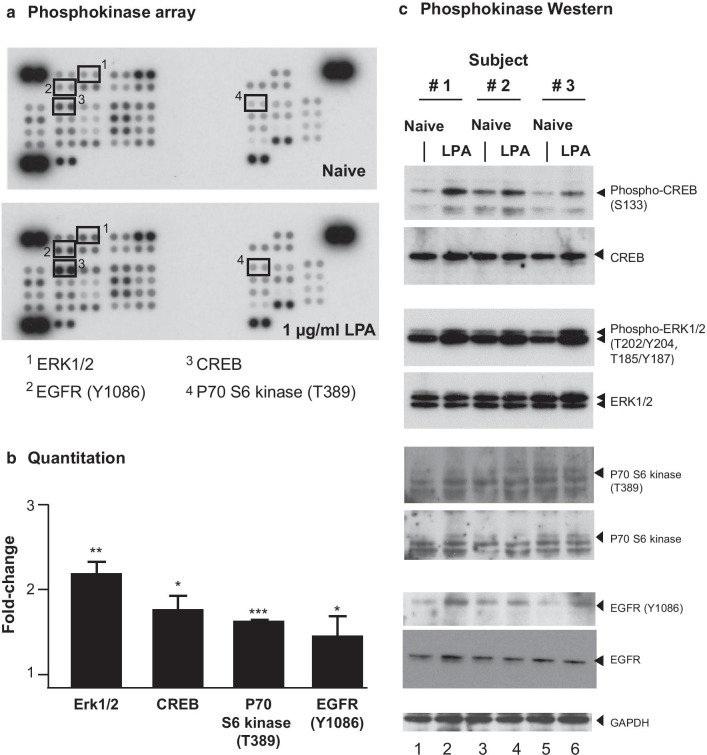
Signaling pathways activated by LPA in SAE BC. Primary basal cells were plated in triplicate in the absence or presence of 1.0 μg/ml LPA for 2.5 min and evaluated for expression of 45 protein kinases. **a** Human phosphokinase array revealing changes in kinase phosphorylation levels following LPA treatment. 1-Erk1/2, 2-EGFR (Y1086), 3-CREB and 4-p70 S6 kinase (T389). **b** Quantification of protein kinase array by Image J software showing top 4 protein kinases with enhanced phosphorylation following LPA treatment. The main pathways stimulated by LPA included Erk1/2, CREB, p70 S6 kinase, and EGFR. Data are expressed as the ratio of the mean signal intensity in the presence of LPA to the mean signal intensity in the absence of LPA of the 3 donors ± SEM. Significance of each set of intensities was determined using a Chi square test. *p < 0.05, **p < 0.01, ***p < 0.001. **c** Western analysis of CREB, ERK1/2, P70 S6 kinase, and EGFR kinase phosphorylation in the absence or presence of LPA confirming phosphorylation of CREB, ERK1/2, and EGFR. Shown is data of BC ± LPA for 3 subjects

The ability of LPA to affect expression of pro-fibrotic factors in small airway basal cells presupposes the presence of LPA receptors and intracellular signaling following ligand-receptor interaction. The presence of LPA receptors (LPARs) and LPA-binding sphingosine-1-phosphate receptors (S1PRs) in small airway basal cells was assessed by quantitative RT-PCR with 18S RNA for normalization using RNA isolated from each of the three donor cell cultures. Receptor expression detected included LPAR1, 2, 3, 5, and 6, and S1PR1, 3, 4, and 5 (Additional file 1: Figure S2). LPAR4 and S1PR2 were not detected. To determine whether LPA interaction with its receptors led to intracellular signaling, SAE BC’s were treated with basal medium (negative control) or basal medium containing 1 μg/ml LPA for 2.5 min. At the conclusion of the incubation, cells were collected by lysis and analyzed using a phospho-kinase array. The data indicated that LPA binding to nonsmoker SAE BC significantly increased levels of phosphorylated cAMP response element binding protein (CREB), ERK1/2 kinase, p70 S6 kinase, and epithelial growth factor receptor (EGFR) phosphorylation compared to the control (Fig. 2a). Quantitation of the intensity of exposure of the films for each kinase demonstrated that signals for phosphorylation of CREB, ERK1/2, p70 S6 kinase and EGFR were all significantly elevated in the LPA-treated sample compared to the naive sample (Fig. 2b). The array contained another 41 potential phosphorylation targets (Additional file 1: Figure S3), but none were upregulated. In order to confirm the increase in phosphorylation of these signaling pathway members, phosphorylated peptides of each kinase were assayed using Western analysis with antibodies that recognized phosphorylation-specific as well as total populations of each target. The Western analysis validated elevated phosphorylation of CREB (S133), ERK1/2 (T202/Y204, T185/Y187), and EFGR (Y1086) in LPA-treated samples compared with naive samples. The phosphorylation of p70 S6 kinase (T389) could not be validated, despite the use of 3 different antiphospho-p70 S6 kinase antibodies (Fig. 2c). As a result, only CREB, ERK1/2 and EGFR were chosen for further investigation.

To evaluate the potential roles of the CREB, ERK1/2, and EGFR signaling in LPA-induced expression of pro-fibrotic growth factors, SAE BC were treated with 1 μg/ml LPA for 3 h in the absence or presence of signaling pathway inhibitors and either collected immediately for RNA analysis or washed and placed in basal medium for 24 h when proteins in the cell culture medium were assessed. To dissect the contributions of different signaling pathways, the 3 h incubation was performed in the absence or presence of 3 signaling pathways: CREB inhibitor 666-15 [35], ERK1/2 inhibitor LY3214996 [36], and EGFR inhibitor AG1478 [37]. The dose of 666-15 and LY3214996 were determined by performing dose response curves to identify the lowest concentration of inhibitor that gave a significant decrease in LPA-induced *CTGF* or *EDN1* gene expression (Additional file 1: Figures S4, S5). The dose of AG1478 was based on prior experience in airway basal cells [38]. The resulting inhibitor doses (200 nM 666-15, 50 nM LY3214996, and 10 µM AG1478) were demonstrated to not evoke cytotoxicity in the basal cells (Additional file 1: Figure S6).

**Fig. 3:**
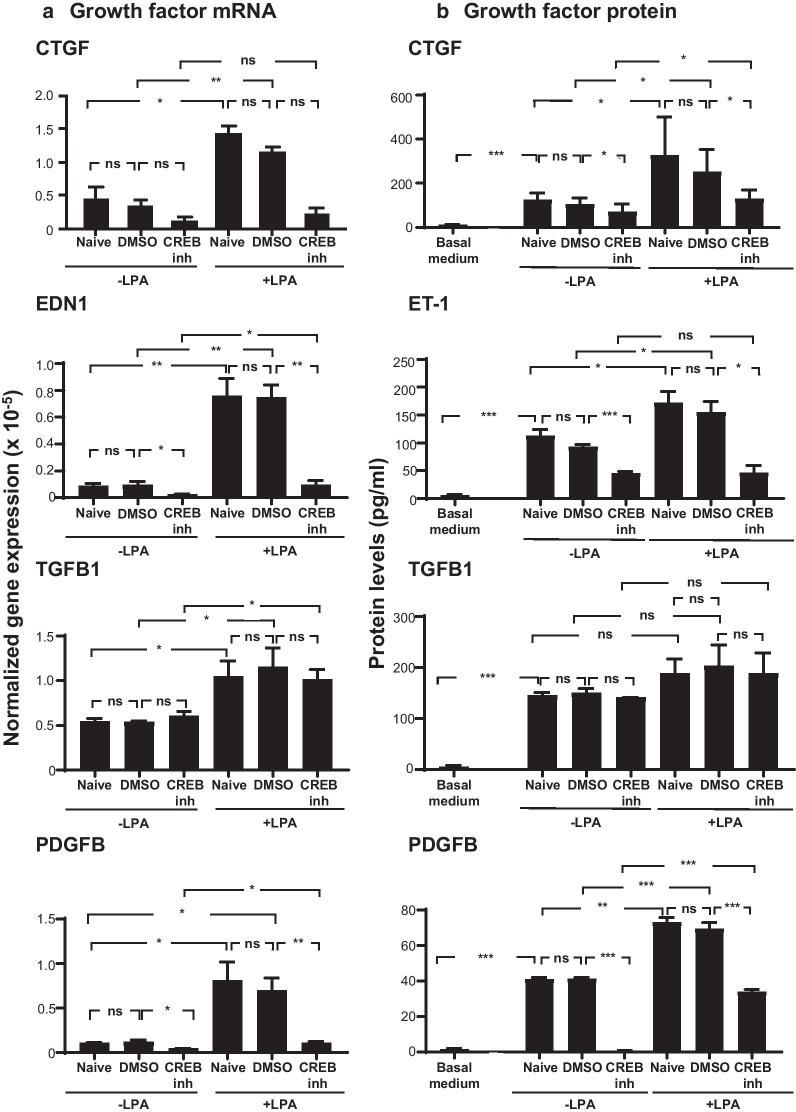
Effect of CREB inhibitor (CREB inh) on growth factor mRNA and protein levels. Primary basal cells from each of three non-smoking donors were plated in triplicate in the presence or absence of 1 μg/ml LPA, 200 nM CREB inhibitor 666-15 and a combination of LPA and CREB inhibitor 666-15. **a** mRNA expression levels. Shown are connective tissue growth factor (*CTGF*), endothelin-1 (*EDN1*), transforming growth factor beta (*TGFB1*) and platelet derived growth factor B (*PDGFB*) mRNA levels determined after 3 h of LPA exposure with and without CREB inhibitor (666-15) using qRT-PCR using 18S RNA to normalize the samples. **b** Protein levels. Shown are protein levels in conditioned media of LPA with and without CREB inhibitor 666-15, LPA with or without CREB inhibitor. After 3 h, the stimuli/inhibitor were removed and cells were incubated with non-supplemented basal media for 24 h. Conditioned media was harvested and evaluated for growth factor levels by ELISA. Data are expressed as the mean value of the 3 donors ± SEM. ND not detected. *p < 0.05, **p < 0.01, ***p < 0.001

Each inhibitor was evaluated for its ability to block LPA-induced changes in pro-fibrotic gene expression and protein secretion in SAE BC from 3 healthy donors. When naive SAE BC were treated with CREB inhibitor 666-15, there was no significant change in *CTGF* mRNA expression. In contrast, the significant elevation of *CTGF* mRNA observed following a 3 h LPA treatment was eliminated in the presence of 666-15 (Fig. 3a). This change was not attributable to the presence of the diluent, DMSO. *EDN1* mRNA levels in both naive and LPA-treated SAE BC were significantly reduced in the presence of 666-15; this inhibitor returned *EDN1* mRNA in LPA-treated samples to baseline levels.

The effect of 666-15 was not universal with respect to pro-fibrotic factors. *TGFB1* mRNA levels were unaffected by 666-15 in the absence or presence of LPA treatment, but *PDGFB* mRNA levels followed the same pattern as *CTGF* and *EDN1* with LPA-elevated levels of mRNA returning to normal when the CREB signaling pathway was blocked (Fig. 3a). Changes in mRNA levels for *CTGF*, *EDN1*, and *PDGFB* were mirrored by changes in the levels of the corresponding proteins, CTGF, ET-1, and PDGF, secreted into the culture medium when evaluated 24 h following the 3 h LPA treatment, while TGFB1 protein levels, like its mRNA, were unchanged by the presence of the CREB inhibitor (Fig. 3b). None of the pro-fibrotic factors were detected in the basal medium.

**Fig. 4:**
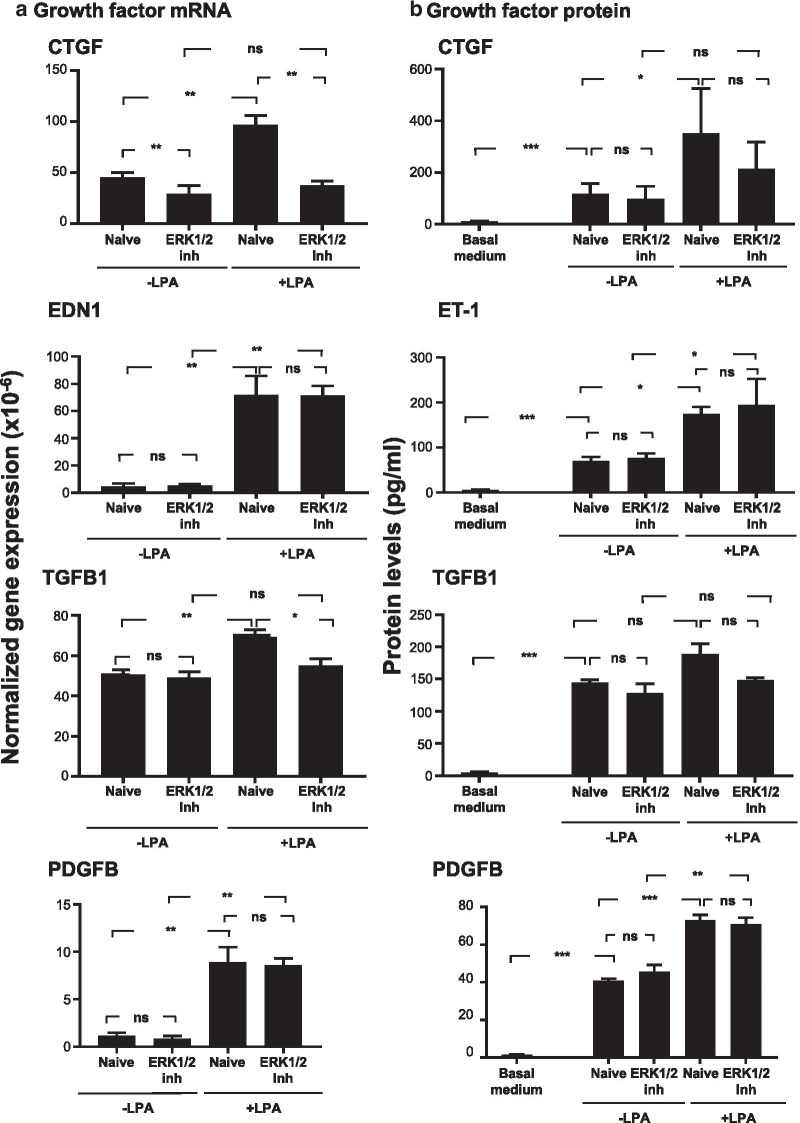
Effect of ERK1/2 inhibitor (ERK1/2 inh) on growth factor expression. Primary basal cells from each of 3 non-smoking donors were plated in triplicate in the presence or absence of 1 μg/ml LPA, 5 µM ERK1/2 inhibitor (LY32149966) and a combination of LPA and ERK1/2 inhibitor (LY32149966). **a** mRNA. Shown are expression levels of connective tissue growth factor (*CTGF*), endothelin-1 (*EDN1*), transforming growth factor beta (*TGFB1*) and platelet derived growth factor B (*PDGFB*) determined after 3 h of LPA exposure with and without ERK1/2 inhibitor (LY32149966) using qRT-PCR using 18S RNA to normalize the samples. **b** Protein. Shown are growth factor levels in the conditioned media. After 3 h of LPA with and without CREB inhibitor (666-15) exposure, LPA with or without ERK1/2 inhibitor was removed and cells were incubated with un-supplemented basal media for 24 h. Conditioned media was harvested and evaluated for growth factor content by ELISA. Data are expressed as the mean value of the 3 donors ± SEM. ND—not detected. *p < 0.05, **p < 0.01, ***p < 0.001

A different pattern of changes was introduced in the presence of ERK1/2 inhibitor, LY3214996. LY3214996 had no significant effect on mRNA levels for pro-fibrotic factors in naive SAE BC, but in LPA-treated BC, LY3214996 was only able to reverse the LPA-induced increase in *CTGF* mRNA, but not the increase in *EDN1* or *PDGFB* mRNAs as observed with the CREB inhibitor (Fig. 4a). LY3214996 was also distinguished from 666-15 in that it was able to reverse the LPA-induced increase in *TGFB1* mRNA, an effect that was not observed with 666-15. At the protein level, no differences were noted in secreted protein levels for any pro-fibrotic factor when comparing naive SAE BC to LY3214996-treated SAE-BC (Fig. 4b). However, the significant reductions in *CTGF* and *TGFB1* mRNA observed with LY3214996 treatment were not reflected in protein levels for those factors where protein levels trended lower but were not significantly different due to inter-donor SAE BC variation (Fig. 4b). ET-1 and PDGFB protein levels were unaffected by the presence of LY3214996 in LPA-treated samples as predicted by *EDN1* and *PDGFB* mRNA levels (Fig. 4b).

**Fig. 5:**
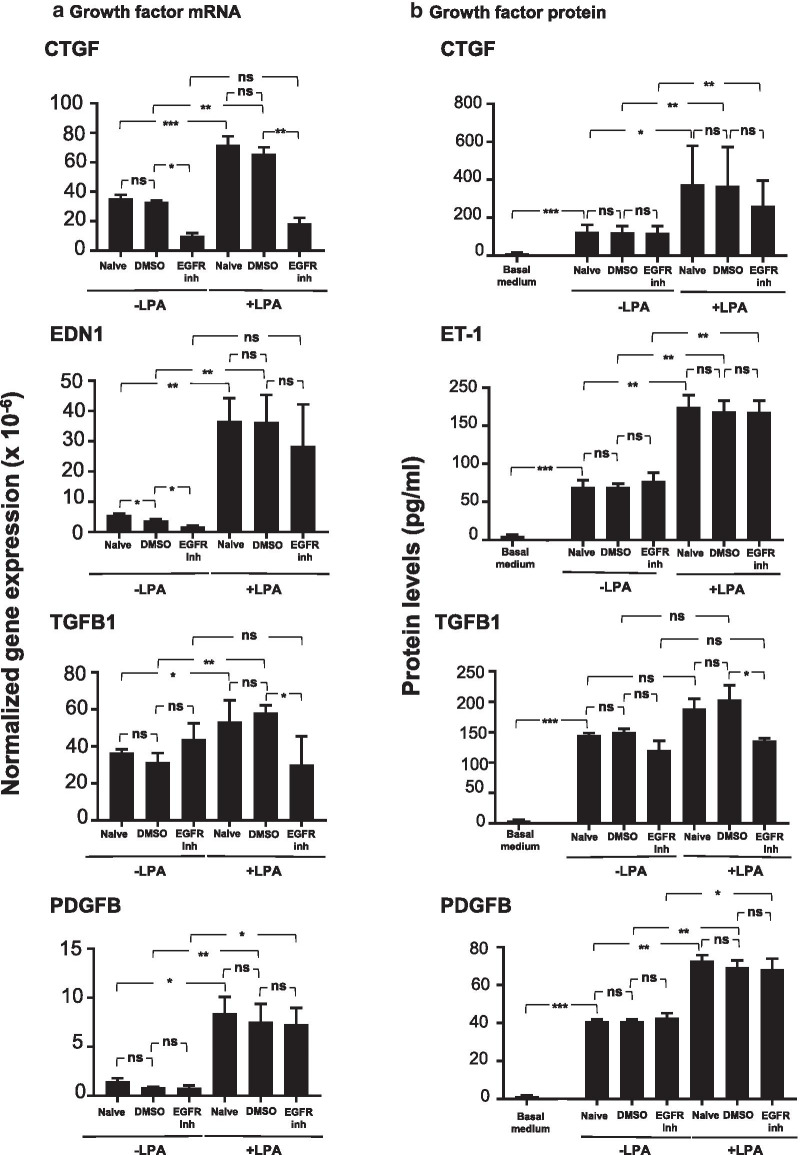
Effect of EGFR inhibitor (EGFR inh) on growth factor mRNA levels. Primary basal cells (passage 3) from each of three non-smoking donors were plated in triplicate in the presence or absence of 1 μg/ml LPA, 10 µM EGFR inhibitor (AG1478), and a combination of LPA and EGFR inhibitor. **a** mRNA expression levels shown are mRNA levels of connective tissue growth factor (*CTGF*), endothelin-1(*EDN1*), transforming growth factor beta (*TGFB1*), and platelet derived growth factor B were determined after 3 h of LPA exposure with and without the inhibitor using qRT-PCR using 18S RNA to normalize the samples. **b** Protein levels in the conditioned media. After 3 h of LPA with and without EGFR inhibitor (AG1478) exposure, LPA with or without inhibitor was removed and cells were incubated with non-supplemented basal media for 24 h. Conditioned media was harvested and evaluated for growth factor content by ELISA. Data are expressed as the mean value of the 3 donors ± SEM. ND—not detected. *p < 0.05, **p < 0.01, ***p < 0.001

Results of the addition of the EGFR inhibitor, AG1478, were very similar to the outcome with the ERK1/2 inhibitor, LY3214996. AG1478 had no effect on *EDN1*, *TGFB1*, or *PDGFB* mRNA in naive SAE BC; however, whereas *CTGF* mRNA was significantly decreased in naive SAE BC by AG1478 treatment (Fig. 5a). AG1478 prevented the LPA-induced elevation of both *CTGF* and *TGFB1* mRNA as observed for the ERK1/2 inhibitor, LY3214996. At the protein level, only TGFB1 levels appeared to be sensitive to AG1478 treatments, showing significant reduction when the inhibitor accompanied LPA treatment (Fig. 5b). No other significant changes in protein levels were induced by AG1478.

### Effect of LPA-treated SAE BC-conditioned Medium on Fibroblast Parameters

**Fig. 6:**
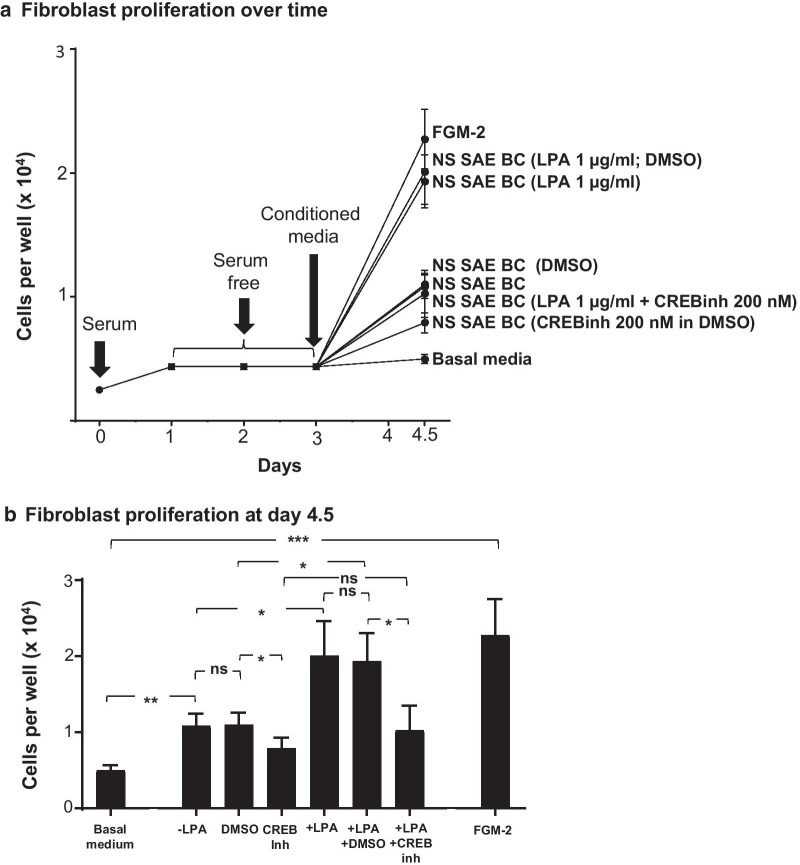
Effect of CREB inhibitor (CREB inh) on fibroblast proliferative response to basal cell conditioned medium. Primary normal human lung fibroblast (NHLF) were plated in triplicate wells and treated with conditioned media from each of 3 non-smoking donors obtained in the presence or absence of 1 μg/ml LPA. For each condition, conditioned medium collected from cultures that were naive or treated with vehicle control (DMSO) or 200 nM CREB inhibitor (666-15) were collected and transferred to NHLF that had been serum starved for 48 h. **a** Fibroblast growth. NHLF were counted after 24 h. Shown is data from BC of a single individual. **b** Summary data showing mean fibroblast cell proliferation exposed to BC conditioned media from 3 donors. Data are expressed as the mean value of the 3 donors ± SEM. *p < 0.05, **p < 0.01, ***p < 0.001

In order to evaluate the pro-fibrotic nature of LPA-induced changes in SAE BC-conditioned medium, SAE BC were treated with 1 μg/ml LPA for 3 h, washed and placed in basal medium for 24 h at which time the “conditioned medium” was collected and transferred onto cultures of normal human lung fibroblasts (NHLF) for assessment of fibroblast fibrotic-related parameters. To assess the effect of conditioned media on fibroblast proliferation, conditioned medium was added to serum-starved cultures of NHLF that had withdrawn from the cell cycle. Positive and negative controls for this experiment included transfer of serum starved NHLF into either FGM-2 medium, an optimized fibroblast growth medium, as a positive control, or transfer into the SAE BC basal medium without exposure to BC cultures (negative control). Day 3 NHLF cultures treated with BC basal medium survived but did not proliferate in the next 36 h while day 3 NHLF cultures treated with FGM-2 medium had approximately 2 cell doublings over the next 36 h (Fig. 6a). When day 3 NHLF were treated with conditioned media from naive BC or BC treated with the DMSO diluent control, the cells underwent a single cell doubling after 36 h, but when the conditioned medium was derived from BC cultures that treated with LPA or LPA plus the DMSO diluent control, NHLF doubled twice within 36 h or treatment (Fig. 6a). When the conditioned medium was derived from LPA cultures that also contained the CREB inhibitor, 666-15, NHLF were only able to double once over the next 36 h as if LPA treatment had not occurred (Fig. 6a, b).

**Fig. 7:**
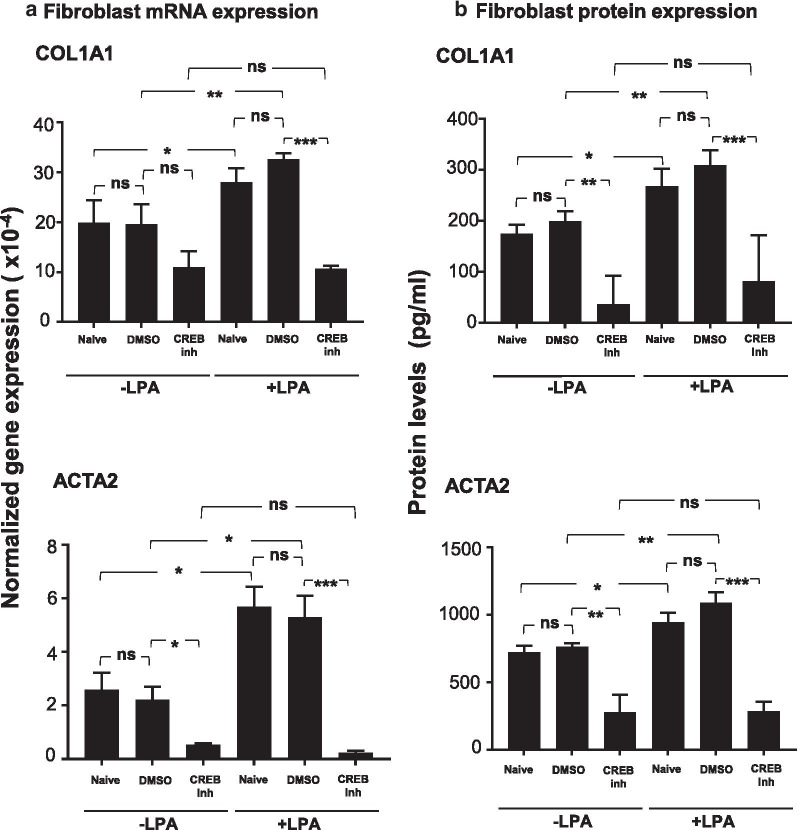
Effect of CREB inhibitor (CREB inh) on fibroblast expression in response to basal cell conditioned medium. Primary normal human lung fibroblasts (NHLF) were treated with conditioned media from each of 3 non-smoking donors obtained in the presence or absence of 1 μg/ml LPA. For each condition, conditioned medium collected from cultures that were naive or treated with vehicle control (DMSO) or 200 nM CREB inhibitor (666-15) were collected and transferred to NHLF. Fibroblast expression of collagen type 1 (*COL1A1*) and smooth muscle actin (*ACTA2*) was assessed after 24 h in conditioned medium. **a** mRNA levels. RNA was harvested and evaluated by qRT-PCR using 18S RNA to normalize the samples. **b** Protein levels. At the same time point, conditioned media was collected and assessed for COL1A1 secretion. Cells were solubilized with a RIPA buffer and evaluated for alpha smooth muscle myosin by ELISA. Data are expressed as the mean value of the 3 donors ± SEM. *p < 0.05, **p < 0.01, ***p < 0.001

In addition to changes in cell proliferation, NHLF were assessed for changes in gene mRNA and protein expression 36 h after exposure to conditioned medium. To test for an effect on extracellular matrix production, quantitative RT-PCR was performed to assess expression of the *COL1A1*. To test changes from fibroblast to myofibroblast phenotype, quantitative RT-PCR was used to assess expression of the *ACTA2* gene that encodes alpha smooth muscle actin (SMA). Both targets were evaluated using conditioned medium from naive and LPA-treated SAE BC in the absence or presence of signaling pathway inhibitors. Both *COL1A1* and *ACTA2* gene expression was detected in NHLF treated with conditioned medium from naive BC and DMSO diluent control cultures. Addition of the CREB inhibitor to naive SAE BC caused a significant decrease in the ability of SAE BC-conditioned medium to support *ACTA2* expression and a trend, but not significant, toward a decrease in *COL1A1* expression. Expression of both markers was increased in conditioned medium from LPA-treated SAE BC cultures and this effect was abrogated by inclusion of CREB inhibitor during LPA treatment (Fig. 7a). Protein levels of both targets evaluated in NHLF cell lysates showed that the collagen I and alpha SMA protein levels tracked closely with their corresponding mRNA levels (Fig. 7b). When the CREB inhibitor was substituted by either ERK1/2 inhibitor, LY3214996, or EGFR inhibitor, AG1478, the reversal of LPA effects was not as dramatic. LY3214996 was unable to prevent the LPA-induced ability of SAE BC-conditioned medium to increase in *COL1A1* or *ACTA2* mRNA while AG1478 partially blocked the increase in *COL1A1* transcription but had no effect on *ACTA2* (Additional file 1: Figure S7).

### Effect of conditioned medium from LPA-treated SAE BC on autotaxin and LPA levels in NHLF cultures

**Fig. 8:**
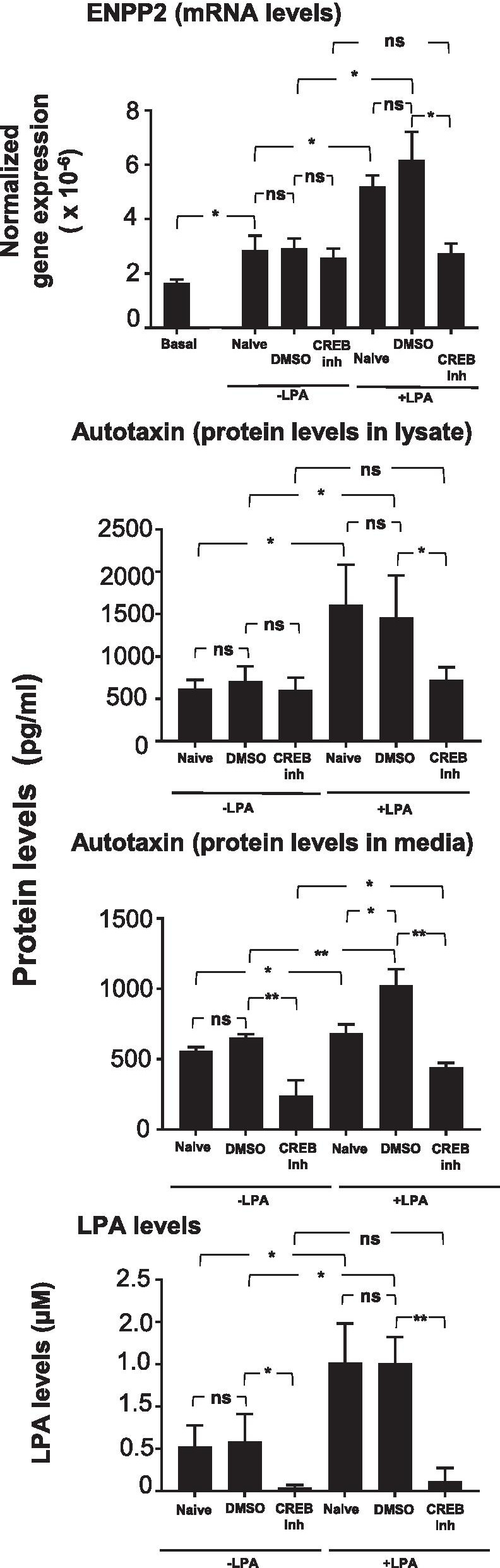
Effect of CREB inhibitor (CREB inh) on autotaxin (*ENPP2*) expression, secretion, and activity in fibroblasts in response to medium conditioned by LPA-treated basal cells. Primary NHLF were treated with conditioned media from each of 3 non-smoking donors obtained in the presence or absence of 1.0 μg/ml LPA. For each condition, conditioned medium collected from cultures that were naive or treated with vehicle control (DMSO) or 200 nM CREB inhibitor (666-15) were collected and transferred to NHLF. Autotaxin, an enzyme responsible for the extracellular production of LPA, in encoded by *ENPP2*. Expression of the *ENPP2* gene was assessed in fibroblasts 24 h after exposure to conditioned medium. Autotaxin protein levels and LPA levels were assessed in cell culture medium 24 h after exposure to conditioned medium. **a** *ENPP2* mRNA levels. mRNA was measured by qRT-PCR using 18S RNA to normalize the samples. **b** Autotaxin protein levels. Protein levels were measured in cell culture medium by ELISA. **c** LPA levels. LPA was measured in cell culture medium by ELISA. Data are expressed as the mean value of the 3 donors ± SEM. *p < 0.05, **p < 0.01, ***p < 0.001

To check whether the conditioned medium from LPA-treated SAE BC affected levels of autotaxin expression in NHLF, RNA, cell lysates and cell culture medium from NHLF cultures were evaluated for expression of *ENPP2* (the gene that encodes autotaxin), autotaxin protein, and LPA levels in the culture medium. The LPA originally used to treat the SAE BC was removed by washing at the end of the 3 h incubation. Control studies using washes from those samples confirmed that bioactivity related to LPA or secreted factors from BC was undetectable (not shown). Transcription of *ENPP2* in NHLF was not affected when treated with SAE BC-conditioned medium including SAE BC-conditioned medium collected in the presence of CREB inhibitor (Fig. 8a). In contrast, *ENPP2* transcription was upregulated when NHLF were treated with SAE BC-conditioned medium that was collected following a 3 h treatment with LPA. This effect was not observed when the conditioned medium was derived from SAE BC treated simultaneously with LPA and CREB inhibitor (Fig. 8a). Autotaxin levels were measured in NHLF cell lysates to determine whether cell-associated autotaxin levels correlated with *ENPP2* expression levels leading to a finding that autotaxin levels followed the same pattern as *ENPP2* expression (Fig. 8b). The level of autotaxin in the NHLF cell culture medium was increased following treatment of NHLF with SAE BC-conditioned medium following LPA treatment, but not when that LPA treatment included CREB inhibitor (Fig. 8c). The elevated level of autotaxin in the cell culture medium of NHLF incubated with conditioned medium from LPA-treated SAE BC translated into a higher level of autotaxin’s enzymatic product, LPA under the same conditions (Fig. 8d). As expected, inclusion of CREB inhibitor in during LPA treatment of SAE BC resulted in conditioned medium that failed to show elevated levels of LPA in the NHLF cultures.

The level of extracellular autotaxin was significantly reduced when NHLF were treated with conditioned medium taken from naive (not LPA-stimulated) SAE BC-conditioned medium that included CREB inhibitor compared with conditioned medium from naïve SAE BC that did not contain CREB inhibitor (Fig. 8c). The reduction of extracellular autotaxin due to SAE BC exposure to CREB inhibitor alone was consistent with the observation that LPA levels were also reduced in NHLF medium following treatment with conditioned medium from SAE BC treated only with CREB inhibitor (Fig. 8d).

## Discussion

**Fig. 9:**
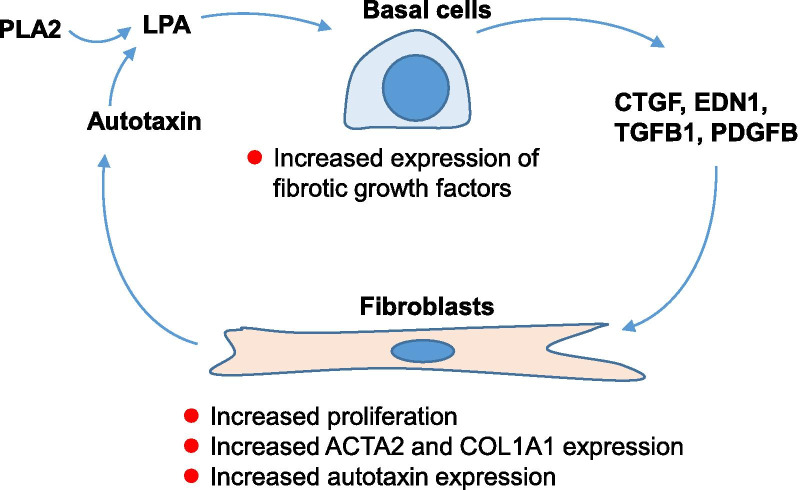
Model for basal cell-fibroblast interaction in IPF. There is a positive feedback relationship in which elevated levels of LPA induce the production of pro-fibrotic factors in basal cells leading to fibrotic changes in fibroblasts including elevated production and secretion of autotaxin, the enzyme that generates LPA. Interruption of the basal cell-fibroblast LPA feedback loop, potentially via inhibition of autotaxin inhibitors, LPAR receptors, CREB-dependent intracellular signaling or any other targets in the LPA signaling pathway, may slow lung fibrosis

The focus of this study was to assess the potential contribution of LPA to the pathogenesis of IPF. SAE BC were evaluated based on the knowledge that the fibrotic foci observed in IPF are comprised of fibroblasts closely apposed to proliferating, undifferentiated epithelial cells with characteristics of SAE BC [2, 5, 8, 39]. The data demonstrates that SAE BC respond to LPA exposure by upregulating expression and secreting elevated levels pro-fibrotic growth factors implicated in lung fibrosis [2, 5, 11–15, 22]. Conditioned medium from LPA-treated SAE BC increased proliferation of lung fibroblasts and induced elevated expression of *COL1A1*, *ACTA2*, and *ENPP2* genes, genes indicative of a pro-fibrotic response in the fibroblasts. Elevated *ENPP2* expression in fibroblasts correlated with elevated autotaxin in the medium of fibroblast cultures and a corresponding increase in LPA levels in the medium of fibroblast cultures. Taken together, the data illustrate a positive feedback loop involving SAE BC and fibroblasts (Fig. 9). This loop could explain how an initial insult in the lung leading to elevated LPA levels could propagate ongoing elevated levels of LPA driving profibrotic conditions, a hallmark of IPF.

There are limitations to the data. The set of experiments employs transfer of conditioned medium as a surrogate for having the cells share the same extracellular milieu as would occur in vivo. The focus was on secreted protein not total transcribed protein. The conditioned medium model enabled the correlation of changes in gene expression with corresponding changes in protein levels in both SAE BC and fibroblasts. Ultimately, it would be ideal to model the intact, functioning feedback loop in vitro providing a means to test hypotheses for interrupting LPA signaling. Thus, the absence of a co-culture model represents a limitation of this study. In characterizing the conditioned medium model system, the focus was on proteins present in the SAE BC-conditioned medium as markers of the output of LPA-treated SAE BC. In maintaining the focus on the content of the conditioned medium, the opportunity to characterize the total change in protein expression, including cell-associated protein, was not pursued, but would have created a more complete picture of the SAE BC response to LPA.

### Role of LPA in pulmonary fibrosis

LPA is produced by the action of two enzymes, phospholipase A2 and autotaxin (a secreted form of phospholipase D) that act on phosphatidylcholine to remove the choline group and one acyl chain resulting in a monoacylphosphoglycerol [25, 40]. LPA has been linked to IPF through several lines of evidence. LPA was found to be elevated in the ELF of mice that had received bleomycin, an experimental model for IPF [24, 28]. Expression of LPAR1 and 2 was implicated since bleomycin-treated mice lacking LPAR1 or LPAR2 were protected from developing lung fibrosis [24, 30]. LPAR1 and autotaxin antagonists were found to block the development of fibrosis in bleomycin-treated mice [28, 31]. Lung epithelial lining fluid from IPF patients has elevated levels of LPA [24].

The CREB signaling pathway was one of 3 signaling pathways to be significantly increase by LPA treatment of SAE BC. The presence of a CREB inhibitor during LPA- treatment of SAE BC prevented LPA-induced expression of *CTGF*, *EDN1*, and *PDGFB*. Inhibitors of ERK1/2 and EGFR pathways failed to block LPA-induced expression of EDN1 or PDGFB although both blocked LPA-induced expression of *CTGF*. Since CREB inhibition covered the broadest range of LPA-stimulated activities, the effect of CREB inhibitor was assessed with respect to changing the pro-fibrotic character of the conditioned medium. Further analysis demonstrated that CREB inhibition blocked the LPA-induced fibroblast proliferation and gene/protein expression, including expression of autotaxin.

Consistent with these observations, conditioned media from LPA-treated SAE BC resulted in elevated levels of autotaxin in the NHLF. The elevated LPA could not have come from the SAE BC-conditioned medium since the original SAE BC cultures were washed 3 times after LPA treatment before the 24 h incubation to collect conditioned medium; the 3rd wash was tested and confirmed to lack pro-fibrotic activity. Furthermore, fibroblasts are known to be a source of autotaxin [29, 41, 42]. The level of autotaxin expression behaved like a marker of pro-fibrotic activity in the NHLF. The CREB inhibitor blocked the elevation of ENPP2 expression and autotaxin levels (both cell associated and secreted). These data suggest that CREB inhibition may have the ability to break the positive feedback cycle by preventing SAE BC from responding to LPA and, thereby, may reduce LPA levels in the lung.

### LPA signaling as a therapeutic target in IPF

The LPA pathway has been an active source of research for anti-fibrotic drugs [43–45]. The observations in the present study that high LPA levels initiate a positive feedback loop provide further support for the importance of this pathway. Potential targets have included LPARs and autotaxin [28, 43, 46]. An LPAR inhibitor had anti-fibrotic results in clinical trials was eventually discontinued due to adverse effects [47]. Two autotaxin inhibitors are currently being developed [46, 48] with hopes that this target might retain the advantages of blocking LPA activity without inducing the same adverse effects as the LPAR inhibitor. The present study suggests that inhibition of the CREB signaling pathway might also prevent LPA signaling in the context of pulmonary fibrosis. CREB inhibitors have received regulatory approval and have been used for the treatment of cancer and as antihelminthics [49–51], and the literature contains examples of systemic application of CREB inhibitors exhibiting anti-fibrotic effects [52, 53]. In summary, in addition to LPARs and autotaxin, CREB signaling may be an additional target for anti-fibrotic drugs.

## Conclusion

Propagation of IPF beyond an initial injury is thought to occur by virtue of one or more positive-feedback signaling loops. The present study shows that an initial, brief exposure of BC to LPA changes BC physiology in a manner that leads to production of pro-fibrotic signaling molecules and a conditioned medium that can go on to induce myofibroblast differentiation in lung fibroblasts. Remarkably, the changes in fibroblast physiology include elevated expression of autotaxin resulting in elevated levels of LPA in the fibroblast-conditioned medium. We conclude that basal cells and fibroblasts sharing a niche in the small airway may drive a pro-fibrotic signaling loop that utilizes LPA signaling, potentially contributing to the pathophysiology of IPF.

## Supplementary Information

**Additional file 1: Table SI.** Study subject demographics. **Figure S1.** Evaluation of growth factor expression in basal cells in the context of a range of LPA levels. Primary small airway basal cells (passage 3) were obtained from each of three healthy, non-smoking donors and plated in triplicate in the presence or absence of 0.1, 1.0, or 10 μg/ml LPA. Cells were evaluated for expression of *CTGF*, *EDN1*, *TGFβ1*, and *PDGFA*, *PDGFB*, and *PDGFC*. After a 3 h of LPA exposure, RNA was harvested and evaluated by qRT-PCR using 18S RNA to normalize the results. Data are expressed as the mean value of the 3 donors ± SE. *p < 0.05, **p < 0.01, ***p < 0.001. **Figure S2**. Expression of LPA receptors in healthy non-smoker BC. Primary small airway basal cells (passage 3) were plated in triplicate and evaluated for expression of lysophosphatidic receptor family members: LPAR1, LPAR2, LPAR3, LPAR4, LPAR5, LPAR6 and sphingosine-1-phosphophate receptor family members: S1PR1, S1PR2, S1PR3, S1PR4, S1PR5. RNA was harvested and evaluated by qRT-PCR using 18S RNA to normalize the results. Data are expressed as the mean value of the 3 donors ± SE. **Figure S3.** Human phospho-kinase array coordinates. Reference spots for the R&D Systems Proteome Profilier Human Phospho-kinase Array including kinases that did not respond significantly to LPA treatment. **Figure S4.** Assessment of SAE BC toxicity following exposure to kinase inhibitors. Basal cell LDH release was assessed using an LDH enzyme activity assay in cell culture medium 24 h after a 4 h exposure to kinase inhibitors. Data are normalized to cell culture volume tested. There was no toxicity observed at the levels of inhibitors used (“NS” – not significant; p > 0.2 compared to naive). **Figure S5.** Effect of ERK1/2, and EGFR inhibitors on fibroblast response to basal cell conditioned medium. Primary normal human lung fibroblasts (NHLF, passage 5) were treated with BC-conditioned media from each of three non-smoking donors obtained in the presence or absence of 1 μg/ml LPA. Conditioned medium was collected from cultures that were naive or treated with 5 µM ERK1/2 inhibitor (LY32149966), or EGFR inhibitor (AG1478). Conditioned medium was then transferred to naive NHLF. After 24 h, expression of collagen type 1 (COL1A1) and smooth muscle actin (ACTA2) in fibroblasts was assessed after 24 h in conditioned medium. RNA was harvested and evaluated by qRT-PCR using 18S RNA to normalize the samples. **A.** Medium collected following treatment with ERK1/2 inhibitor. **B.** Medium collected following treatment with P70 S6 kinase inhibitor. **C.** Medium collected following treatment with EGFR inhibitor. Data are expressed as the mean value of the 3 donors ± SE. *p < 0.05, **p < 0.01, ***p < 0.001. **Figure S6.** Evaluation of growth factor expression in basal cells in the context of the presence or absence of 1 μg/ml LPA and a range of CREB inhibitor (666-15) concentrations. Primary small airway basal cells (passage 3) were obtained from each of three healthy, non-smoking donors and plated in triplicate presence or absence of 1 μg/ml LPA, and a combination of LPA and 200 nM, 1 μM or 2 μM CREB inhibitor (666-15). Cells were evaluated for *CTGF*, *EDN1*, *TGFβ1*, and *PDGFB*. After a 3 h of LPA exposure, RNA was harvested and evaluated by qRT-PCR using 18S RNA to normalize the results. Data are expressed as the mean value of the 3 donors ± SE. *p < 0.05, **p < 0.01, ***p < 0.001. **Figure S7.** Evaluation of growth factor expression in basal cells in the context of the presence or absence of 1 μg/ml LPA and a range of ERK inhibitor (LY32149966) concentrations. Primary small airway basal cells (passage 3) were obtained from each of three healthy, non-smoking donors and plated in triplicate presence or absence of 1 μg/ml LPA, and a combination of LPA and 5 nM, 50 nM, 500 nM, 1 μM, 5 μM or 10 μM ERK1/2 inhibitor (LY32149966). Cells were evaluated for *CTGF*, *EDN1*, *TGFβ1*, and *PDGFB*. After a 3 h of LPA exposure, RNA was harvested and evaluated by qRT-PCR using 18S RNA to normalize the results. Data are expressed as the mean value of the 3 donors ± SE. *p < 0.05, **p < 0.01, ***p < 0.001.

## Data Availability

Primary basal cells used in this study have limited expansion capacity. Contact the corresponding author regarding availability.

## References

[1] Fulmer JD, Roberts WC, von Gal ER, Crystal RG (1977). Small airways in idiopathic pulmonary fibrosis. Comparison of morphologic and physiologic observations. J Clin Invest.

[2] Crystal RG, Bitterman PB, Mossman B, Schwarz MI, Sheppard D, Almasy L, Chapman HA, Friedman SL, King TE, Leinwand LA (2002). Future research directions in idiopathic pulmonary fibrosis: summary of a National Heart, Lung, and Blood Institute working group. Am J Respir Crit Care Med.

[3] Raghu G, Collard HR, Egan JJ, Martinez FJ, Behr J, Brown KK, Colby TV, Cordier JF, Flaherty KR, Lasky JA (2011). An official ATS/ERS/JRS/ALAT statement: idiopathic pulmonary fibrosis: evidence-based guidelines for diagnosis and management. Am J Respir Crit Care Med.

[4] Kim HJ, Perlman D, Tomic R (2015). Natural history of idiopathic pulmonary fibrosis. Respir Med.

[5] Martinez FJ, Collard HR, Pardo A, Raghu G, Richeldi L, Selman M, Swigris JJ, Taniguchi H, Wells AU (2017). Idiopathic pulmonary fibrosis. Nat Rev Dis Primers.

[6] Lederer DJ, Martinez FJ (2018). Idiopathic pulmonary fibrosis. N Engl J Med.

[7] Crystal RG, Fulmer JD, Baum BJ, Bernardo J, Bradley KH, Bruel SD, Elson NA, Fells GA, Ferrans VJ, Gadek JE (1978). Cells, collagen and idiopathic pulmonary fibrosis. Lung.

[8] Scotton CJ, Chambers RC (2007). Molecular targets in pulmonary fibrosis: the myofibroblast in focus. Chest.

[9] Habiel DM, Hogaboam C (2014). Heterogeneity in fibroblast proliferation and survival in idiopathic pulmonary fibrosis. Front Pharmacol.

[10] Evans CM, Fingerlin TE, Schwarz MI, Lynch D, Kurche J, Warg L, Yang IV, Schwartz DA (2016). Idiopathic pulmonary fibrosis: a genetic disease that involves mucociliary dysfunction of the peripheral airways. Physiol Rev.

[11] Bonner JC (2004). Regulation of PDGF and its receptors in fibrotic diseases. Cytokine Growth Factor Rev.

[12] Horowitz JC, Thannickal VJ (2006). Epithelial-mesenchymal interactions in pulmonary fibrosis. Semin Respir Crit Care Med.

[13] Abraham D (2008). Role of endothelin in lung fibrosis. Eur Respir Rev.

[14] Fonseca C, Abraham D, Renzoni EA (2011). Endothelin in pulmonary fibrosis. Am J Respir Cell Mol Biol.

[15] Richeldi L, Fernandez Perez ER, Costabel U, Albera C, Lederer DJ, Flaherty KR, Ettinger N, Perez R, Scholand MB, Goldin J (2020). Pamrevlumab, an anti-connective tissue growth factor therapy, for idiopathic pulmonary fibrosis (PRAISE): a phase 2, randomised, double-blind, placebo-controlled trial. Lancet Respir Med.

[16] Chilosi M, Zamo A, Doglioni C, Reghellin D, Lestani M, Montagna L, Pedron S, Ennas MG, Cancellieri A, Murer B, Poletti V (2006). Migratory marker expression in fibroblast foci of idiopathic pulmonary fibrosis. Respir Res.

[17] Gokey JJ, Snowball J, Sridharan A, Speth JP, Black KE, Hariri LP, Perl AT, Xu Y, Whitsett JA (2018). MEG3 is increased in idiopathic pulmonary fibrosis and regulates epithelial cell differentiation. JCI Insight.

[18] Gokey JJ, Sridharan A, Xu Y, Green J, Carraro G, Stripp BR, Perl AT, Whitsett JA (2018). Active epithelial Hippo signaling in idiopathic pulmonary fibrosis. JCI Insight.

[19] Xu Y, Mizuno T, Sridharan A, Du Y, Guo M, Tang J, Wikenheiser-Brokamp KA, Perl AT, Funari VA, Gokey JJ (2016). Single-cell RNA sequencing identifies diverse roles of epithelial cells in idiopathic pulmonary fibrosis. JCI Insight.

[20] Reyfman PA, Walter JM, Joshi N, Anekalla KR, McQuattie-Pimentel AC, Chiu S, Fernandez R, Akbarpour M, Chen CI, Ren Z (2019). Single-cell transcriptomic analysis of human lung provides insights into the pathobiology of pulmonary fibrosis. Am J Respir Crit Care Med.

[21] Carraro G, Mulay A, Yao C, Mizuno T, Konda B, Petrov M, Lafkas D, Arron JR, Hogaboam CM, Chen P (2020). Single cell reconstruction of human basal cell diversity in normal and IPF lung. Am J Respir Crit Care Med.

[22] Sakai N, Tager AM (2013). Fibrosis of two: Epithelial cell-fibroblast interactions in pulmonary fibrosis. Biochim Biophys Acta.

[23] Wolters PJ, Collard HR, Jones KD (2014). Pathogenesis of idiopathic pulmonary fibrosis. Annu Rev Pathol.

[24] Tager AM, LaCamera P, Shea BS, Campanella GS, Selman M, Zhao Z, Polosukhin V, Wain J, Karimi-Shah BA, Kim ND (2008). The lysophosphatidic acid receptor LPA1 links pulmonary fibrosis to lung injury by mediating fibroblast recruitment and vascular leak. Nat Med.

[25] Shea BS, Tager AM (2012). Role of the lysophospholipid mediators lysophosphatidic acid and sphingosine 1-phosphate in lung fibrosis. Proc Am Thorac Soc.

[26] Tager AM (2012). Autotaxin emerges as a therapeutic target for idiopathic pulmonary fibrosis: limiting fibrosis by limiting lysophosphatidic acid synthesis. Am J Respir Cell Mol Biol.

[27] Valdes-Rives SA, Gonzalez-Arenas A (2017). Autotaxin-lysophosphatidic acid: from inflammation to cancer development. Mediators Inflamm.

[28] Swaney JS, Chapman C, Correa LD, Stebbins KJ, Bundey RA, Prodanovich PC, Fagan P, Baccei CS, Santini AM, Hutchinson JH (2010). A novel, orally active LPA(1) receptor antagonist inhibits lung fibrosis in the mouse bleomycin model. Br J Pharmacol.

[29] Oikonomou N, Mouratis MA, Tzouvelekis A, Kaffe E, Valavanis C, Vilaras G, Karameris A, Prestwich GD, Bouros D, Aidinis V (2012). Pulmonary autotaxin expression contributes to the pathogenesis of pulmonary fibrosis. Am J Respir Cell Mol Biol.

[30] Huang LS, Fu P, Patel P, Harijith A, Sun T, Zhao Y, Garcia JG, Chun J, Natarajan V (2013). Lysophosphatidic acid receptor-2 deficiency confers protection against bleomycin-induced lung injury and fibrosis in mice. Am J Respir Cell Mol Biol.

[31] Ninou I, Kaffe E, Müller S, Budd DC, Stevenson CS, Ullmer C, Aidinis V (2018). Pharmacologic targeting of the ATX/LPA axis attenuates bleomycin-induced pulmonary fibrosis. Pulm Pharmacol Ther.

[32] Harvey BG, Heguy A, Leopold PL, Carolan BJ, Ferris B, Crystal RG (2007). Modification of gene expression of the small airway epithelium in response to cigarette smoking. J Mol Med (Berl).

[33] Li D, Liu H, Li Y, Yang M, Qu C, Zhang Y, Liu Y, Zhang X (2014). Identification of suitable endogenous control genes for quantitative RT-PCR analysis of miRNA in bovine solid tissues. Mol Biol Rep.

[34] Rennard SI, Basset G, Lecossier D, O'Donnell KM, Pinkston P, Martin PG, Crystal RG (1985). Estimation of volume of epithelial lining fluid recovered by lavage using urea as marker of dilution. J Appl Physiol.

[35] Li BX, Gardner R, Xue C, Qian DZ, Xie F, Thomas G, Kazmierczak SC, Habecker BA, Xiao X (2016). Systemic inhibition of CREB is well-tolerated in vivo. Sci Rep.

[36] Sheng X, Li J, Zhang C, Zhao L, Guo L, Xu T, Jin J, Wu M, Xia Y (2019). α-Mangostin promotes apoptosis of human rheumatoid arthritis fibroblast-like synoviocytes by reactive oxygen species-dependent activation of ERK1/2 mitogen-activated protein kinase. J Cell Biochem.

[37] Lee FT, Mountain AJ, Kelly MP, Hall C, Rigopoulos A, Johns TG, Smyth FE, Brechbiel MW, Nice EC, Burgess AW, Scott AM (2005). Enhanced efficacy of radioimmunotherapy with 90Y-CHX-A''-DTPA-hu3S193 by inhibition of epidermal growth factor receptor (EGFR) signaling with EGFR tyrosine kinase inhibitor AG1478. Clin Cancer Res.

[38] Zuo WL, Yang J, Gomi K, Chao I, Crystal RG, Shaykhiev R (2017). EGF-amphiregulin interplay in airway stem/progenitor cells links the pathogenesis of smoking-induced lesions in the human airway epithelium. Stem Cells.

[39] Richeldi L, Collard HR, Jones MG (2017). Idiopathic pulmonary fibrosis. Lancet.

[40] Aoki J (2004). Mechanisms of lysophosphatidic acid production. Semin Cell Dev Biol.

[41] Santos AN, Riemann D, Santos AN, Kehlen A, Thiele K, Langner J (1996). Treatment of fibroblast-like synoviocytes with IFN-gamma results in the down-regulation of autotaxin mRNA. Biochem Biophys Res Commun.

[42] Kehlen A, Lauterbach R, Santos AN, Thiele K, Kabisch U, Weber E, Riemann D, Langner J (2001). IL-1 beta- and IL-4-induced down-regulation of autotaxin mRNA and PC-1 in fibroblast-like synoviocytes of patients with rheumatoid arthritis (RA). Clin Exp Immunol.

[43] Kihara Y, Mizuno H, Chun J (2015). Lysophospholipid receptors in drug discovery. Exp Cell Res.

[44] Suryadevara V, Ramchandran R, Kamp DW, Natarajan V (2020). Lipid mediators regulate pulmonary fibrosis: potential mechanisms and signaling pathways. Int J Mol Sci.

[45] Zulfikar S, Mulholland S, Adamali H, Barratt SL (2020). Inhibitors of the autotaxin-lysophosphatidic acid axis and their potential in the treatment of interstitial lung disease: current perspectives. Clin Pharmacol.

[46] Kolb M, Bonella F, Wollin L (2017). Therapeutic targets in idiopathic pulmonary fibrosis. Respir Med.

[47] Palmer SM, Snyder L, Todd JL, Soule B, Christian R, Anstrom K, Luo Y, Gagnon R, Rosen G (2018). Randomized, double-blind, placebo-controlled, phase 2 trial of BMS-986020, a lysophosphatidic acid receptor antagonist for the treatment of idiopathic pulmonary fibrosis. Chest.

[48] Nikolaou A, Kokotou MG, Limnios D, Psarra A, Kokotos G (2017). Autotaxin inhibitors: a patent review (2012–2016). Expert Opin Ther Pat.

[49] Xiao X, Li BX, Mitton B, Ikeda A, Sakamoto KM (2010). Targeting CREB for cancer therapy: friend or foe. Curr Cancer Drug Targets.

[50] Chae HD, Cox N, Dahl GV, Lacayo NJ, Davis KL, Capolicchio S, Smith M, Sakamoto KM (2018). Niclosamide suppresses acute myeloid leukemia cell proliferation through inhibition of CREB-dependent signaling pathways. Oncotarget.

[51] Chen W, Mook RA, Premont RT, Wang J (2018). Niclosamide: beyond an antihelminthic drug. Cell Signal.

[52] Henderson WR, Chi EY, Ye X, Nguyen C, Tien YT, Zhou B, Borok Z, Knight DA, Kahn M (2010). Inhibition of Wnt/beta-catenin/CREB binding protein (CBP) signaling reverses pulmonary fibrosis. Proc Natl Acad Sci U S A.

[53] Hirakawa T, Nasu K, Miyabe S, Kouji H, Katoh A, Uemura N, Narahara H (2019). beta-catenin signaling inhibitors ICG-001 and C-82 improve fibrosis in preclinical models of endometriosis. Sci Rep.

