# Supplementary material for: CREB-dependent LPA-induced signaling initiates a pro-fibrotic feedback loop between small airway basal cells and fibroblasts
Source: Respir Res. 2021 Apr 1;22:97. doi: 10.1186/s12931-021-01677-0 (PMC8015171; doi:10.1186/s12931-021-01677-0)
Supplement: Supplementary file 1 — Additional file 1: Table SI. Study subject demographics. Figure S1. Evaluation of growth factor expression in basal cells in the context of a range of LPA levels. Primary small airway basal cells (passage 3) were obtained from each of three healthy, non-smoking donors and plated in triplicate in the presence or absence of 0.1, 1.0, or 10 μg/ml LPA. Cells were evaluated for expression of CTGF, EDN1, TGFβ1, and PDGFA, PDGFB, and PDGFC. After a 3 h of LPA exposure, RNA was harvested and evaluated by qRT-PCR using 18S RNA to normalize the results. Data are expressed as the mean value of the 3 donors ± SE. *p < 0.05, **p < 0.01, ***p < 0.001. Figure S2. Expression of LPA receptors in healthy non-smoker BC. Primary small airway basal cells (passage 3) were plated in triplicate and evaluated for expression of lysophosphatidic receptor family members: LPAR1, LPAR2, LPAR3, LPAR4, LPAR5, LPAR6 and sphingosine-1-phosphophate receptor family members: S1PR1, S1PR2, S1PR3, S1PR4, S1PR5. RNA was harvested and evaluated by qRT-PCR using 18S RNA to normalize the results. Data are expressed as the mean value of the 3 donors ± SE. Figure S3. Human phospho-kinase array coordinates. Reference spots for the R&D Systems Proteome Profilier Human Phospho-kinase Array including kinases that did not respond significantly to LPA treatment. Figure S4. Assessment of SAE BC toxicity following exposure to kinase inhibitors. Basal cell LDH release was assessed using an LDH enzyme activity assay in cell culture medium 24 h after a 4 h exposure to kinase inhibitors. Data are normalized to cell culture volume tested. There was no toxicity observed at the levels of inhibitors used (“NS” – not significant; p > 0.2 compared to naive). Figure S5. Effect of ERK1/2, and EGFR inhibitors on fibroblast response to basal cell conditioned medium. Primary normal human lung fibroblasts (NHLF, passage 5) were treated with BC-conditioned media from each of three non-smoking donors obtained in the presence or absence [file 12931_2021_1677_MOESM1_ESM.docx]

**Supplemental Methods**

**LPA Stimulation of Small Airway Basal Cells**

To study the dose dependent effect of lysophosphatidic acid (LPA) on basal cells (BC), BC were plated in PneumaCult ExPlus complete medium (StemCell Technologies, Cambridge, MA). LPA was prepared as a 1 mM stock in distilled, deionized molecular grade water (Ambion, Austin, TX). After 24 hr, BC were washed twice with PBS, once with unsupplemented ExPlus medium, and incubated with unsupplemented ExPlus medium in the absence (control) of presence of LPA in a dose response (0.1, 1.0, 10.0 $\mu$g/ml; Echelon Bioscience, Salt Lake City, UT) for 3 hr. After a 3 hr incubation, BC’s were harvested with Trizol (Invitrogen) for RNA isolation.

**Evaluation of LPA Receptor Expression in Basal Cells**

The level of expression of LPA receptor genes, including LPAR1, LPAR2 LPAR3, LPAR4, LPAR5, LPAR6 and sphingosine 1 phosphate receptors including S1PR1, S1PR2, S1PR3, S1PR4, S1PR5 were determined using qRT-PCR as described in the main text. RNA was isolated from passage 3 basal cells during expansion in ExPlus complete medium. Probes for qRT-PCR were purchased from Applied Biosystems (Foster City, CA) and included: LPAR1 (Hs00173500_m1), LPAR2 (Hs01109356_m1), LPAR3 (Hs00173857_m1), LPAR4 (Hs00271072_s1), LPAR5 (Hs00252675_s1), LPAR6 (Hs00271758_s1), S1PR1(Hs01922614_s1), S1PR2(ARU64HX_m1)), S1PR3 (Hs00245464_s1), S1PR4 (Hs02330084_s1), S1PR5 (Hs00928195_s1).

**CREB Inhibition and Erk1/2 Inhibition (Dose Response) of LPA Stimulation of SAE BC**

To study the dose dependent effect of CREB inhibition on BC stimulated with an optimal concentration of LPA (1 $\mu$g/ml, determined in the prior dose-response experiment), BC were plated in PneumaCult ExPlus complete medium. CREB inhibitor 666-15 (Eschelon Biosciences) was prepared as a 10 mM stock solution in dimethylsulfoxide (DMSO; Sigma, St. Louis, MO). After 24 hr, BC were washed twice with PBS, once with unsupplemented ExPlus medium, and incubated with unsupplemented ExPlus medium in the absence (control) or presence of LPA (1$\mu$g/ml) $\pm$ CREB inhibitor 666-15 (Eschelon Biosciences) in a dose response for 3 hr (0.2, 1.0, or 2.0 $\mu$M in .005% DMSO, were used as controls for each CREB inhibitor concentration) $\pm$ ERK1/2 inhibitor LY3214996 (Med Chem Express, Monmouth Junction, NJ) in a dose response for 3 hr 0.005, 0.05, 0.5, 1.0, 5.0 or 10.0 $\mu$M in 0.01% DMSO, were used as controls for each CREB inhibitor concentration) After a 3 hr incubation, BC’s were harvested with Trizol for RNA isolation.

**Toxicity Levels of Each Inhibitor**

To study the cytotoxic effects of CREB, ERK1/2, and EGFR inhibition on BC, BC were plated, treated, and harvested for conditioned media as described above. Conditioned media were then analyzed for the presence of LDH levels using the Roche LDH cytotoxic assay kit (Sigma) according to the manufacturer’s instructions.

**Supplemental Table I. Study subject demographics**

| **Sample^1^** | **Status^2^** | **Basal cell passage** | **Region^3^** | **Age** | **Sex** | **Ethnicity^4^** |
| --- | --- | --- | --- | --- | --- | --- |
| DGM-13577 | Healthy nonsmoker | 3 | DRLL/DLLL | 41 | F | C |
| DGM-01657 | Healthy nonsmoker | 3 | DRLL/DLLL | 55 | M | C |
| DGM-13767 | Healthy nonsmoker | 3 | DRLL/DLLL | 55 | M | C |

^1^ De-identified sample number

^2^ Smoking status confirmed by analysis of smoking metabolites; undetectable levels of urine nicotine, urine cotinine; pulmonary function tests within the normal range

^3^ Distal right lower lobe (DRLL) or distal left lower lobe (DLLL)

^4^ Caucasian (C)

**Supplemental Figure 1.** Evaluation of growth factor expression in basal cells in the context of a range of LPA levels. Primary small airway basal cells (passage 3) were obtained from each of three healthy, non-smoking donors and plated in triplicate in the presence or absence of 0.1, 1.0, or 10 μg/ml LPA. Cells were evaluated for expression of *CTGF*, *EDN1*, *TGFβ1*, and *PDGFA*, *PDGFB*, and *PDGFC*. After a 3 hr of LPA exposure, RNA was harvested and evaluated by qRT-PCR using 18S RNA to normalize the results. Data are expressed as the mean value of the 3 donors ± SE. *p<0.05, **p<0.01, ***p<0.001.

**Supplemental Figure 2**. Expression of LPA receptors in healthy non-smoker BC. Primary small airway basal cells (passage 3) were plated in triplicate and evaluated for expression of lysophosphatidic receptor family members: LPAR1, LPAR2, LPAR3, LPAR4, LPAR5, LPAR6 and sphingosine-1-phosphophate receptor family members: S1PR1, S1PR2, S1PR3, S1PR4, S1PR5. RNA was harvested and evaluated by qRT-PCR using 18S RNA to normalize the results. Data are expressed as the mean value of the 3 donors ± SE.

**Supplemental Figure 3.** Human phospho-kinase array coordinates. Reference spots for the R&D Systems Proteome Profilier Human Phospho-kinase Array including kinases that did not respond significantly to LPA treatment.

**Supplemental Figure 4.** Assessment of SAE BC toxicity following exposure to kinase inhibitors. Basal cell LDH release was assessed using an LDH enzyme activity assay in cell culture medium 24 hr after a 4 hr exposure to kinase inhibitors. Data are normalized to cell culture volume tested. There was no toxicity observed at the levels of inhibitors used (“NS” – not significant; p > 0.2 compared to naive).

**Supplemental Figure 5.** Effect of ERK1/2, and EGFR inhibitors on fibroblast response to basal cell conditioned medium. Primary normal human lung fibroblasts (NHLF, passage 5) were treated with BC-conditioned media from each of three non-smoking donors obtained in the presence or absence of 1 μg/ml LPA. Conditioned medium was collected from cultures that were naive or treated with 5µM ERK1/2 inhibitor (LY32149966), or EGFR inhibitor (AG1478). Conditioned medium was then transferred to naive NHLF. After 24 hr, expression of collagen type 1 (COL1A1) and smooth muscle actin (ACTA2) in fibroblasts was assessed after 24 hr in conditioned medium. RNA was harvested and evaluated by qRT-PCR using 18S RNA to normalize the samples. **A.** Medium collected following treatment with ERK1/2 inhibitor. **B.** Medium collected following treatment with P70 S6 kinase inhibitor. **C.** Medium collected following treatment with EGFR inhibitor. Data are expressed as the mean value of the 3 donors ± SE. *p<0.05, **p<0.01, ***p<0.001.

**Supplemental Figure 6.** Evaluation of growth factor expression in basal cells in the context of the presence or absence of 1 μg/ml LPA and a range of CREB inhibitor (666-15) concentrations. Primary small airway basal cells (passage 3) were obtained from each of three healthy, non-smoking donors and plated in triplicate presence or absence of 1μg/ml LPA, and a combination of LPA and 200 nM, 1 μM or 2 μM CREB inhibitor (666-15). Cells were evaluated for *CTGF*, *EDN1*, *TGFβ1*, and *PDGFB*. After a 3 hr of LPA exposure, RNA was harvested and evaluated by qRT-PCR using 18S RNA to normalize the results. Data are expressed as the mean value of the 3 donors ± SE. *p<0.05, **p<0.01, ***p<0.001.

**Supplemental Figure 7.** Evaluation of growth factor expression in basal cells in the context of the presence or absence of 1 μg/ml LPA and a range of ERK inhibitor (LY32149966) concentrations. Primary small airway basal cells (passage 3) were obtained from each of three healthy, non-smoking donors and plated in triplicate presence or absence of 1 μg/ml LPA, and a combination of LPA and 5 nM, 50 nM, 500 nM, 1 μM, 5 μM or 10 μM ERK1/2 inhibitor (LY32149966). Cells were evaluated for *CTGF*, *EDN1*, *TGFβ1*, and *PDGFB*. After a 3 hr of LPA exposure, RNA was harvested and evaluated by qRT-PCR using 18S RNA to normalize the results. Data are expressed as the mean value of the 3 donors ± SE. *p<0.05, **p<0.01, ***p<0.001.
